# Endophthalmitis: a bibliometric study and visualization analysis from 1993 to 2023

**DOI:** 10.3389/fcimb.2024.1355397

**Published:** 2024-07-16

**Authors:** Xiangyu Fu, Wenyu Du, Ling Huang, Xiang Ren, Danian Chen

**Affiliations:** ^1^ Department of Ophthalmology, West China Hospital, Sichuan University, Chengdu, China; ^2^ Research Laboratory of Ophthalmology and Vision Sciences, Eye Research Institute, West China Hospital, Sichuan University, Chengdu, China

**Keywords:** post-cataract endophthalmitis, post-injection, post-traumatic, endogenous endophthalmitis, bacterial, fungal, Citespace, VOSviewer

## Abstract

**Aims:**

This study is designed to generalize and depict the research hotspots of endophthalmitis through bibliometric methods and software and analyze the evolutive tendency of the work on this severe disease over the past 30 years.

**Methods:**

This study employed a rigorous bibliometric approach. We identified all endophthalmitis-related literature by conducting a comprehensive search of the Science Citation Index Expanded database under the Web of Science Core Collection. The data was then analyzed and visualized using CiteSpace and VOSviewer, two widely recognized software tools in the field of bibliometrics. CiteSpace was used to analyze the country distributions, dual map overlay of journals, keyword bursts, and co-cited references. VOSviewer was employed to describe the authors and co-cited authors, the journals, the co-cited journals, and the keywords co-occurrence network. This robust methodology ensures the reliability and validity of the study’s findings.

**Results:**

A total of 2960 publications, including 2695 articles and 265 reviews, were included in this bibliometric study. There has been no shortage of endophthalmitis-related publications since 1993, with an apparent upward trend during recent years. Possible correlations with the COVID-19 pandemic are also analyzed. These studies were finished by 11,048 authors from 75 countries worldwide, with the United States in the lead. In the keyword co-occurrence network, except for the endophthalmitis term, cataract surgery becomes the keyword with the highest frequency. Different categories of endophthalmitis, including postoperative, post-injection, post-traumatic, and endogenous endophthalmitis, and antibacterial and anti-inflammatory therapies of infectious endophthalmitis, are discussed by categories. From the perspective of the timeline, postoperative and post-injection endophthalmitis were the dominant forms before and after the year 2000, respectively. Co-citation analyses reveal that the Endophthalmitis Vitrectomy Study (EVS) conducted in 1995 provides pivotal guidance for later research. Diverse pathogenic bacteria (e.g., Coagulase-negative *Staphylococci*, *Propionibacterium acnes*, *Viridians Streptococci*, and *Bacillus cereus*) or fungi (e.g., *Candida*, *Aspergillus*, and *Fusarium*) contribute to varying treatment principles and clinical prognosis, which should be taken seriously. In addition, intravitreal and intracameral antibiotics are the mainstay for treating and preventing infectious endophthalmitis, respectively.

**Conclusion:**

Our bibliometric analysis provides an overview of dynamic evolution and structural relationships in the research field of endophthalmitis. The displayed hotspots and developmental directions have reference values for future investigation.

## 1 Introduction

Endophthalmitis describes inflammation within the eyeball, usually involving the vitreous cavity and aqueous humor in the anterior chamber. Still, adjacent intraocular tissues, such as the choroid and retina, are frequently affected (Lodha et al., 2022). As one of the most dreaded ocular conditions, endophthalmitis is a highly vision-threatening complication that can even lead to blindness in severe cases (Kunkler et al., 2022; Tang et al., 2023). Although this severe complication is uncommon, for example, the estimated incidence of acute-onset endophthalmitis after cataract surgery is 0.02%-0.21%, it still places an incalculable economic burden on individuals and society, given the high prevalence of cataracts and the widespread application of ocular surgeries such as cataract extraction. Various causes contribute to different types of endophthalmitis. Infectious and non-infectious endophthalmitis can be recognized depending on the presence of infection. The latter contains sterile uveitis, phacoanaphylactic endophthalmitis, and sympathetic ophthalmia. Sterile uveitis is possibly induced by surgical trauma, retention of lens fragments or foreign bodies, adverse drug reactions, and immune responses (Bhagat et al., 2011; Fu et al., 2021). Phacoanaphylactic endophthalmitis, also known as lens-induced endophthalmitis, occurs when the lens capsule ruptures and is more common in the early stage of extracapsular cataract extraction (ECCE) (Mardelli and Mehanna, 2007). Sympathetic ophthalmia refers to bilateral granulomatous uveitis following penetrating trauma or intraocular surgery of one eye, resulting from a T cell-mediated autoimmune reaction after injury to the uveal tract (Court et al., 2019).

Unless otherwise stated, the endophthalmitis term in the narrow sense usually refers specifically to infectious endophthalmitis, which is more likely to emerge and attract attention in clinical settings. Identifying and classifying the causes of infectious endophthalmitis are particularly crucial, as they correspond to diverse pathogenic microorganisms and distinct management strategies (Ness, 2018). Infectious endophthalmitis can be divided into bacterial and fungal endophthalmitis (Durand, 2017) due to the different types of pathogenic microbes. Symptoms of bacterial endophthalmitis vary from relatively painless anterior chamber inflammation with coagulase-negative *Staphylococci* led by *Staphylococcus epidermidis* (Ermis et al., 2005) to delayed persistent intraocular infections caused by *Propionibacterium acnes* (Huynh and Johnson, 2006) and even to explosive eye and orbital involvement induced by *Bacillus cereus* (Rishi et al., 2013). *Candida albicans* and *Aspergillus* are the main causative pathogens that trigger fungal endophthalmitis with poor visual prognosis (Haseeb et al., 2021). Regarding the infectious route, exogenous and endogenous endophthalmitis can be further distinguished, among which the former covers more common postoperative (principally following cataract surgery) (Arshinoff and Bastianelli, 2011), post-injection (Vanderbeek et al., 2015), and post-traumatic (Bhagat et al., 2011) endophthalmitis. Conversely, endogenous endophthalmitis results from the hematogenous spread of systemic infections, in which pathogenic organisms cross the blood-eye barrier and multiply within the eye. Blood culture is the gold standard for diagnosing endogenous endophthalmitis (Jackson et al., 2003).

Exploring the pathogenic mechanisms and effective therapeutic regimens of endophthalmitis has been a hot spot in eye research for a long time. Classical prospective findings obtained from the Endophthalmitis Vitrectomy Study (EVS) (Vine et al., 1995) and the European Society of Cataract & Refractive Surgeons (ESCRS) multicenter study (Barry and Grp, 2007) have revealed the clinical benefits of therapeutic vitrectomy and prophylactic antibiotics. Experimental endophthalmitis models have also helped in elucidating pathogenic mechanisms. Since the eye is an immune-privilege organ, microorganisms entering the eye can replicate largely unhindered by the immune system. Toxins and cell wall components such as lipopolysaccharides and peptidoglycan fragments produced by bacteria can lead to loss of retinal function. Secretion of pro-inflammatory factors by resident immune cells, the increase of blood-ocular barrier permeability, and the recruitment of phagocytic inflammatory cells jointly contribute to structural disruption, photoreceptor cell apoptosis, and significant inflammatory responses in the eye (Callegan et al., 2002; Lefevre et al., 2012).

Given the complexity of this disease and the diversity of research, there is an urgent need to systematically sort out the research relevant to endophthalmitis, summarize the current mainstream research directions, and display the future development trends in this field, which is currently lacking. The bibliometric study is a powerful tool for achieving these goals and is widely employed in literature analysis. Bibliometric analysis offers a quantitative method to review and investigate the existing references in a particular field. It provides access to familiarity with this area’s research structure and framework (Guler et al., 2016). Furthermore, the visualization atlas generated by bibliometric software can supplement the analytical results, help to vividly interpret the data, and visualize the research focus, which aims to form a clear knowledge context and sufficient literature reference for subsequent research (Gu et al., 2021; Fu et al., 2023). Therefore, this study is designed to generalize and depict the research hotspots of endophthalmitis through bibliometric methods and analyze the evolutive tendency of the work on this severe disease over the past 30 years.

## 2 Materials and methods

### 2.1 Search strategies and data collection

**Figure 1:**
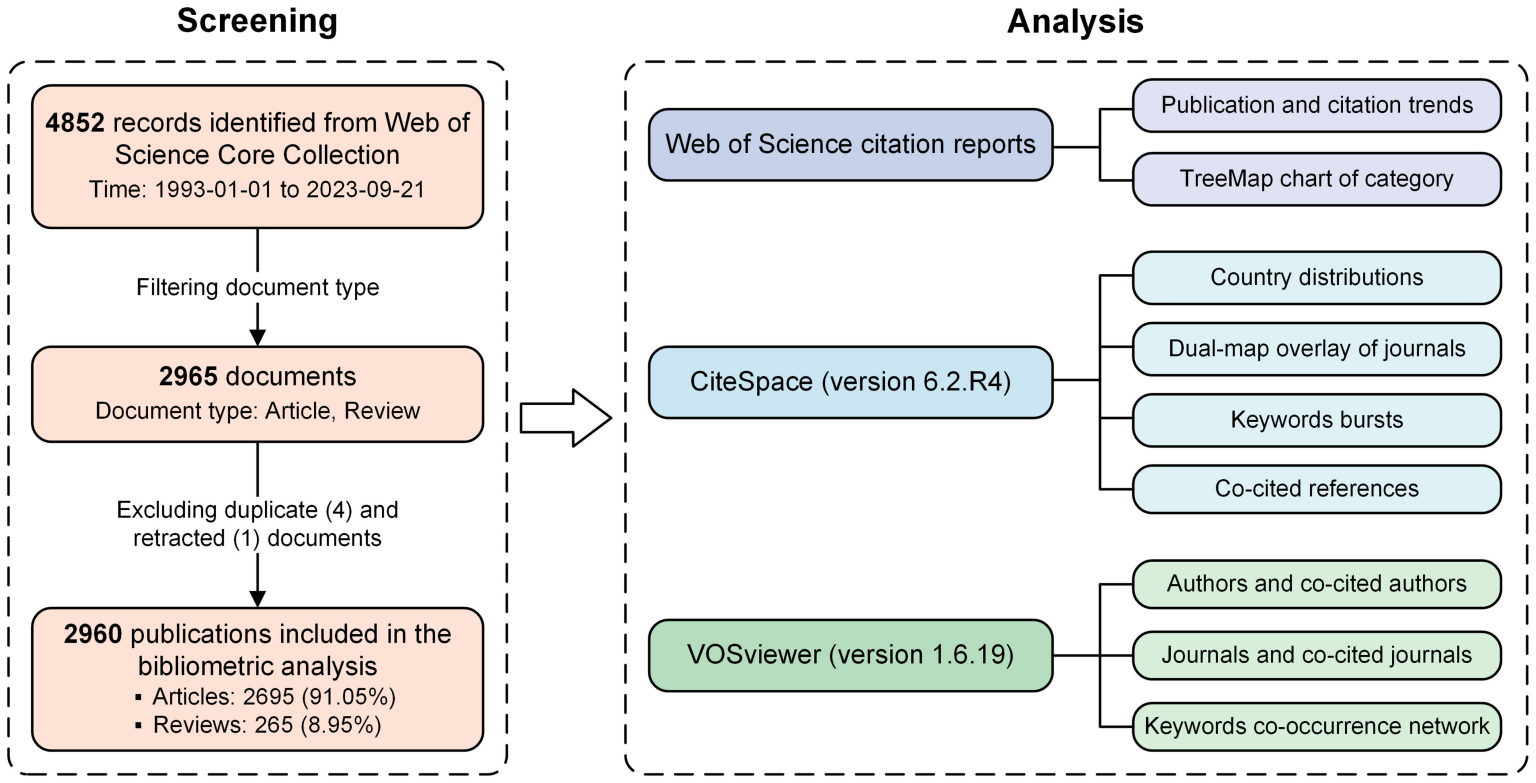
The flow chart of literature screening and analysis in the bibliometric study.

We identified all endophthalmitis-related literature by searching the Science Citation Index (SCI)-Expanded database under the Web of Science Core Collection (WOSCC). All our searches were completed on September 21, 2023, to prevent data bias due to the literature updates. Publications with the term “endophthalmitis” in the title or in both the abstract and keyword lists were considered desirable, and the document type was limited to “articles” and “review articles” covering the period from 1993 to 2003. The specific search strategies are as follows: (TI=(“Endophthalmitis”) OR (AB=(“Endophthalmitis”) AND AK=(“Endophthalmitis”))) AND (Documents type: Articles, Review articles) AND (Timespan: 1993-01-01 to 2023-09-21). By limiting the timespan and filtering document types, 2965 records were identified from the WOSCC. After excluding four duplicate articles and one retracted paper, 2960 publications, including 2695 articles and 265 reviews, were included in this bibliometric study (**Figure 1**). Eligible records were saved and exported as plain text files covering information such as titles, authors, keywords, institutions, countries, published journals, references, and citations.

### 2.2 Bibliometric analysis

Exported data were imported into CiteSpace version 6.2.R4 (Drexel University, Philadelphia, United States) (Chen, 2006) and VOSviewer version 1.6.19 (Leiden University, Leiden, Netherlands) (Van Eck and Waltman, 2010) for the bibliometric analysis. Potentially duplicates were eliminated by the “Remove Duplicates” function in CiteSpace. Then, the synonyms of terms in some parts, such as countries, co-cited journals, and keywords, were merged for more precise explanations. The citation reports from the WOS database supplied the publication and citation trends and the TreeMap chart of research categories from 1993 to 2023 (**Figure 1**). CiteSpace provided analysis and visualization of the country distributions, dual-map overlay of journals, keyword bursts, and co-cited references. As one of the most mainstream bibliometric software, CiteSpace conducts reference co-citation analysis based on a similarity algorithm, which is applied to obtain cluster view and timeline view in time slices to clearly outline the process and historical span of endophthalmitis evolution in the time dimension, and plot the development trends of related research (**Figure 1**). VOSviewer was employed to describe the authors and co-cited authors, the journals and co-cited journals, and the keywords co-occurrence network. According to the standardized method of probability theory, the co-occurrence analysis can distinguish categories of the keywords and display the connections between keywords through clusters with different colors to provide a more precise overview (**Figure 1**).

## 3 Results

### 3.1 The publication and citation trends

**Figure 2:**
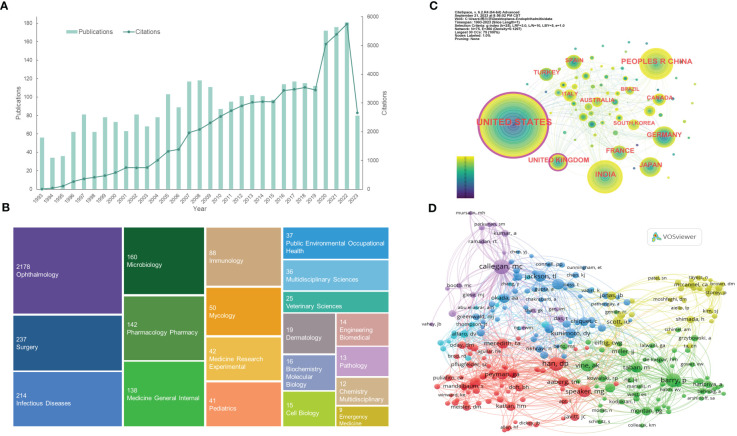
Distribution of publications and citations from different years, categories, countries, and authors. **(A)** The citation report of the publication and citation trends from 1993 to 2023. **(B)** The first 20 research categories belong to the publications. **(C)** Country distributions of the publications. Purple rings on the periphery mean a high centrality. **(D)** Visualization of the co-cited authors. VOSviewer automatically classified co-authors with over 45 citations into five sections (the purple, blue, red, green, and yellow sections, respectively). From: CiteSpace, v. 6.2.R4 (64-bit) Advance. From: VOSviewer.

To some extent, the number of publications and citations can reflect the speed of progression in a particular research field. The publication and citation trends of endophthalmitis-related research are presented in **Figure 2A**. Over the past three decades, the tendency of publications can be broadly divided into three phases. From 1993 to 2009, the number of studies fluctuated but increased overall, averaging around 77 articles published yearly. Even though the number of researches decreased slightly in 2010, which probably correlates with the introduction of anti-vascular endothelial growth factor (anti-VEGF) medications into clinical use (Mezad-Koursh et al., 2010; Inman and Anderson, 2011), it rose steadily over the next decade (2010-2019). Since 2020, the production of endophthalmitis-related articles has increased explosively due to the COVID-19 pandemic, maintaining about 175 publications annually, where endophthalmitis cases in the context of COVID-19 were generally reported and discussed (Mirghorbani et al., 2022; Markan et al., 2023). Similarly, the number of citations has grown steadily year by year, with a dramatic change in 2020. Especially in 2022, 181 papers and 5750 citations came out, reaching the climax (**Figure 2A**). The 2960 publications are cited 64,625 times (an average of 21.83 citations per publication) and 39,312 times without self-citation (an average of 13.28 citations per publication) in the SCI-Expanded database. This result indicates that endophthalmitis has been the focus of study for ophthalmologists and researchers in different periods.

### 3.2 Analysis of research categories

The WOS classifies the search results into 70 categories, with **Figure 2B** showing the first 20. From this TreeMap chart, ophthalmology (2178, 73.6%), surgery (237, 8.0%), infectious diseases (214, 7.2%), microbiology (160, 5.4%), and pharmacology pharmacy (142, 4.8%) are the top five categories. Analysis of different categories can reveal the emphases of various studies. Since endophthalmitis is an ophthalmic disease, the ophthalmology accounts for the vast majority. Surgery term represents relatively common postoperative (e.g., cataract surgery, glaucoma filtering surgery) endophthalmitis and vitrectomy, one of the effective treatments for endophthalmitis. Moreover, the categories of infectious diseases, microbiology, immunology, and mycology reflect that infectious endophthalmitis is caused by a variety of microorganisms and induced inflammatory responses. Bacteria or fungi with different virulence are likely to be closely related to the disease prognosis. Likewise, the pharmacology pharmacy classification emphasizes the importance of pharmacotherapy, especially the selection of antibiotic and antifungal agents with varied ranges of antimicrobial profiles (**Figure 2B**).

### 3.3 Analysis of leading countries

A total of 75 countries participated in the research of endophthalmitis, with **Table 1** listing the top 6 country distributions. Among them, the United States (1001, 33.818%) contributed nearly one-third of the attention and the highest citations, showing a significant quantitative advantage, followed by India (315, 10.642%) and the People’s Republic of China (302, 10.203%) with about 10% studies each (**Table 1**). In the distribution network shown in **Figure 2C**, it is surprising that, although a few publications are from the United Kingdom, this country has a high centrality like the United States, with purple rings around the nodes. Centrality, or betweenness centrality, is an indicator of research impact. It suggests that studies from these two countries may be necessary to the topic or connect different sections of the whole field.

**Table 1:** Top 6 country distributions of publications.

Rank	Country	Centrality	Counts (%)	Citations
1	United States	0.36	1001 (33.818)	33551
2	India	0.08	315 (10.642)	4519
3	Peoples R China	0.08	302 (10.203)	4367
4	Germany	0.09	169 (5.709)	2313
5	France	0.01	154 (5.203)	2192
6	United Kingdom	0.16	146 (4.932)	4307

### 3.4 Analysis of authors and co-cited authors

About 11,048 authors are involved in endophthalmitis-related studies, and the top 10 authors and co-cited authors can be seen in **Table 2**. Consistent with the analysis of leading countries, half of the authors and the vast majority of co-authors in **Table 2** are from the United States, reflecting its outstanding leadership. The top two authors, Flynn HW, and Miller D, are both from the Bascom Palmer Eye Institute and are far ahead in the number of collaborative publications. The following four Indian authors, Sharma S, Das T, Joseph J, and Dave VP, come from the same institution (LV Prasad Eye Institute) and have published dozens of papers. Regarding the listed co-cited authors, it can be discovered that both Han DP and Vine AK are principal investigators in the EVS conducted in 1995 (Vine et al., 1995), which is a well-known multicenter randomized clinical trial funded by the National Eye Institute (NEI) of the United States. Barry P, the research chairman, led a multicenter study accomplished by the ESCRS endophthalmitis study group in 2007 (Barry and Grp, 2007), while Peyman GA was a consultant for the Traumatic Endophthalmitis Trial (TET) also in 2007 (Soheilian et al., 2007). Equally striking, two American authors, Callegan MC, and Scott IU, rank highly on the lists of both authors and co-cited authors (**Table 2**).

**Table 2:** Top 10 authors and co-cited authors.

Rank	Author	Country	Counts (%)	Co-cited author	Country	Citation counts
1	Flynn HW	United States	114 (3.851)	Callegan MC	United States	511
2	Miller D	United States	74 (2.500)	Han DP	United States	446
3	Sharma S	India	56 (1.892)	Barry P	Ireland	439
4	Das T	India	52 (1.757)	Vine AK	United States	362
5	Joseph J	India	43 (1.453)	Speaker MG	United States	334
6	Callegan MC	United States	42 (1.419)	Jackson TL	United Kingdom	294
7	Scott IU	United States	40 (1.351)	Durand ML	United States	291
8	Kumar A	United States	37 (1.250)	Peyman GA	United States	278
9	Dave VP	India	37 (1.250)	Taban M	United States	265
10	Pathengay A	India	30 (1.014)	Scott IU	United States	265

The nodes formed by VOSviewer display co-cited authors with over 45 citations who are divided into several sections in various colors (**Figure 2D**). The purple section, centered on Callegan MC, is dedicated to interpreting the pathogenesis of experimental bacterial endophthalmitis, such as bacterial-host interactions. Represented by Jackson TL, Durand ML, and Okada AA, the blue area focuses more on endogenous endophthalmitis with bacterial or fungal infections. Additionally, Han DP, Speaker MG, and Peyman GA, expressed as prominent red nodes, emphasize the prevention and therapies for endophthalmitis. Besides, the remaining green and yellow parts are relevant to postoperative endophthalmitis. The difference is that the green part mainly covers post-cataract endophthalmitis, while the yellow one pays attention to endophthalmitis that occurs after pars plana vitrectomy (PPV). These connections between the co-cited authors reveal endophthalmitis’s knowledge base and research directions.

### 3.5 Analysis of journals and co-cited journals

**Figure 3:**
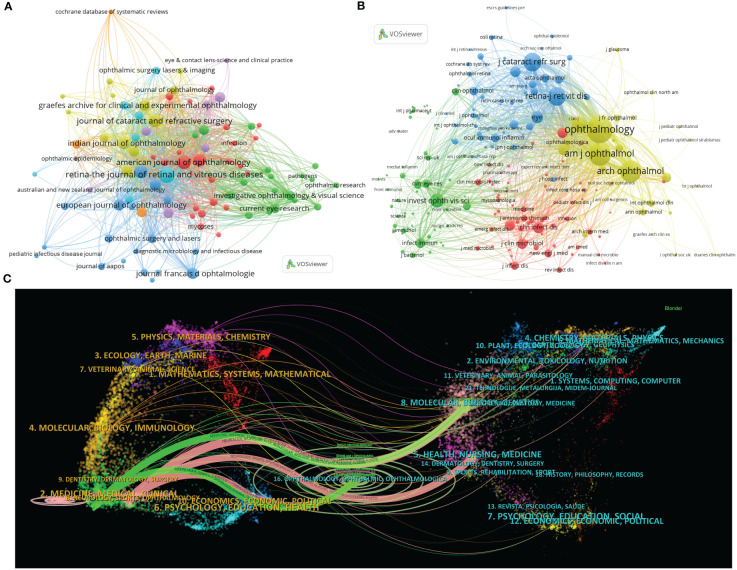
Distribution of publications and citations from different journals. Visualization maps of the journals **(A)** and co-cited journals **(B)**. Journals with more publications or higher co-citation frequency are symbolized as the larger nodes. **(C)** The dual-map overlay of journals reveals the connections between publications and citations, with dots representing citing journals on the left and cited journals on the right so that the citation relationships are depicted as colored lines from the left to the right. From: VOSviewer.

The data collected in this analysis has been published in 394 academic journals, chiefly including *Retina-The Journal of Retinal and Vitreous Diseases* (*Retina*) (217, 7.331%), *American Journal of Ophthalmology* (*AJO*) (150, 5.068%), *Journal of Cataract and Refractive Surgery* (*JCRS*) (128, 4.324%), *Ophthalmology* (122, 4.122%), and *Ocular Immunology and Inflammation* (114, 3.851%) (**Table 3**; **Figure 3A**). They are all well-known ophthalmology or immunology journals.

**Table 3:** Top 11 journals and co-cited journals.

Rank	Journal	Counts (%)	JCR (2023)	Co-cited journal	Citation counts	JCR (2023)
1	*Retina-The Journal of Retinal and Vitreous Diseases*	217 (7.331)	Q2	*Ophthalmology*	9268	Q1
2	*American Journal of Ophthalmology*	150 (5.068)	Q1	*American Journal of Ophthalmology*	6321	Q1
3	*Journal of Cataract and Refractive Surgery*	128 (4.324)	Q1	*Archives of Ophthalmology (JAMA Ophthalmology)*	5566	Q1
4	*Ophthalmology*	122 (4.122)	Q1	*Retina-The Journal of Retinal and Vitreous Diseases*	3687	Q2
5	*Ocular Immunology and Inflammation*	114 (3.851)	Q2	*Journal of Cataract and Refractive Surgery*	3684	Q1
6	*Indian Journal of Ophthalmology*	89 (3.007)	Q2	*British Journal of Ophthalmology*	2652	Q1
7	*Graefes Archive for Clinical and Experimental Ophthalmology*	78 (2.635)	Q2	*Investigative Ophthalmology & Visual Science*	1982	Q1
8	*Journal Francais D Ophtalmologie*	78 (2.635)	Q3	*Eye*	1362	Q1
9	*European Journal of Ophthalmology*	73 (2.466)	Q3	*Clinical Infectious Diseases*	1168	Q1
10	*Eye*	68 (2.297)	Q1	*Survey of Ophthalmology*	1149	Q1
11	*British Journal of Ophthalmology*	64 (2.162)	Q1	*Graefes Archive for Clinical and Experimental Ophthalmology*	1073	Q2

Q1, Quartile 1 of JCR 2023.

The co-citation analysis can also help identify highly influential journals in the field. The top 11 co-cited journals, which have been cited more than 1,000 times, are listed in **Table 3**. Multiple major ophthalmology journals are involved in the list, which highly overlaps with the summary of the most published ones. Among them, *Ophthalmology* is the most frequently co-cited journal with 9,268 citations, followed by *AJO* (6,321 times), *Archives of Ophthalmology* (the predecessor of *JAMA Ophthalmology*) (5,566 times), *Retina* (3,687 times), and *JCRS* (3,684 times).

Moreover, journals with over 30 citation counts are chosen and automatically divided into four clusters in the visualization analysis, as shown in **Figure 3B**. The higher the co-citation frequency of a journal, the larger the corresponding node. Specifically, the yellow cluster features clinical ophthalmic journals such as *Ophthalmology*, *AJO*, and *Archives of Ophthalmology*, symbolizing the frontier of ophthalmic clinical research. Several other ophthalmology journals, like *Retina* and *JCRS*, are included in the blue cluster, corresponding to certain sub-directions in ophthalmology. The green zone primarily comprises *Investigative Ophthalmology & Visual Science* (*IOVS*), a distinguished journal for publishing experimental ophthalmic studies, and academic publications including *Current Eye Research*, *Science*, and *Nature*. In addition, the nodes marked in red are on behalf of clinical journals related to infection and microbiology, with *Clinical Infectious Diseases* and *Journal of Clinical Microbiology* as the representative journals.

Importantly, impact factor (IF) is a widely recognized indicator to weigh a journal’s core influence. Interestingly, using IF 2023 as the standard, *Ophthalmology* has the highest IF (13.1) both in the top 11 journals and co-cited journals. Furthermore, according to the journal citation reports (JCR) in 2023 (Clarivate, United Kingdom), the majority of the leading journals and co-cited journals are located in Quartile 1 (Q1) or Q2 (**Table 3**).

Simultaneously, CiteSpace can link the citing and cited bibliographical categories, thus demonstrating this one-to-one correspondence in the dual-map overlay of journals (Chen et al., 2014). Citation relations are depicted as broad colored strokes starting from the left side representing the citing journals and pointing to the right, which denotes the cited ones. There are five main thick lines, including two green paths from the Medicine/Medical/Clinical category and three pink pathways from Neurology/Sports/Ophthalmology-related journals (**Figure 3C**). Of note, the green and pink trajectories intersect at sections of Ophthalmology/Ophthalmic/Ophthalmologica and Molecular/Biology/Genetics, indicating that ophthalmic clinical trials and basic experimental studies jointly support the entire research field of endophthalmitis (**Figure 3C**).

### 3.6 Analysis of co-occurring keywords and burst terms

Keyword analysis is vital because research hotspots and focuses in the field often come from the co-occurrence and bursts of the keywords. Some similar terms were amalgamated before the formal analysis, including synonyms (e.g., “ocular injuries” and “eye injuries”), different expressions (e.g., “contact lens” and “contact-lenses”), and singular and plural forms (e.g., “risk-factors” and “risk-factor”). **Table 4** provides access to the top 25 keywords, which can be divided into two categories. The first category describes the causes or classification of endophthalmitis, such as “cataract surgery”, “intravitreal injection”, “endogenous”, and “fungal”. The second category concerns strategies for the prevention and treatment (both medical and surgical therapy), including but not limited to terms like “povidone-iodine”, “antibiotics”, “vancomycin”, “amphotericin-b”, and “vitrectomy”.

**Table 4:** Top 25 keywords.

Rank	Keywords	Counts
1	endophthalmitis	1298
2	cataract surgery	459
3	bacterial endophthalmitis	417
4	post-operative endophthalmitis	403
5	vitrectomy	386
6	infection	322
7	outcomes	269
8	management	257
9	risk-factors	257
10	intravitreal injection	198
11	endogenous endophthalmitis	196
12	prophylaxis	194
13	surgery	173
14	fungal endophthalmitis	169
15	infectious endophthalmitis	156
16	antibiotics	154
17	diagnosis	114
18	povidone-iodine	113
19	keratitis	108
20	bevacizumab (avastin)	104
21	spectrum	103
22	vancomycin	103
23	injection	102
24	eye	101
25	amphotericin-b	99

**Figure 4:**
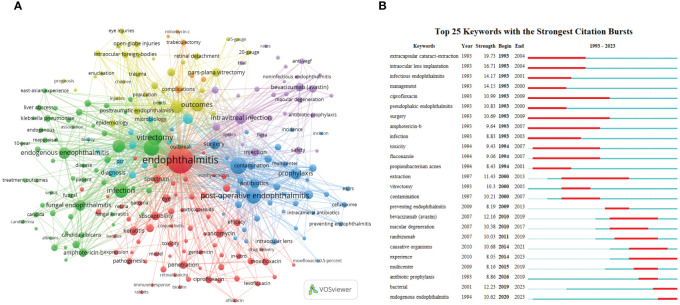
The main keywords. **(A)** Keyword co-occurrence networks. The node size indicates the frequency of keyword occurrence, and the lines connecting nodes represent the strength of the link between keywords. **(B)** The top keywords with the most robust citation bursts. The long blue line depicts the whole timeline (1993-2023), and the short red line indicates the burst period of specific keywords.

VOSviewer was used to generate a co-occurrence network of keywords, exhibited in **Figure 4A**, and intuitively divided keywords into several parts, similar to the analyses of co-cited authors and journals. On the one hand, the blue, purple, yellow, and green areas represent the research themes on different types of endophthalmitis, respectively.

First, phacoemulsification and intraocular lens implantation in cataract surgery are identified as the leading causes of postoperative endophthalmitis (blue section). Some options for the prophylaxis of endophthalmitis, such as the preoperative use of povidone-iodine to avoid the contamination of conjunctival bacteria, have been proposed and are likely to be effective (Fintelmann and Naseri, 2010). Second, with the increasing prevalence of fundus neovascular diseases like proliferative diabetic retinopathy (PDR) and wet macular degeneration in recent years, the demand for intravitreal injection of antiangiogenic drugs such as bevacizumab (Avastin) has gradually risen, resulting in possible complicated post-injection endophthalmitis (purple nodes). Subsequently, the yellow area focuses on post-traumatic endophthalmitis, principally caused by open globe injuries. The presence or absence of intraocular foreign bodies (IOFB), their nature, and the degree of retinal detachment are critical factors in determining the prognosis of visual acuity, and most cases often require vitrectomy (Durand, 2013). Moreover, there is a relative increase in the proportion of adolescents with this type of ocular inflammation. Finally, rare endogenous endophthalmitis (5%-15% of all endophthalmitis cases) is discussed in the green cluster. Among them, endogenous bacterial endophthalmitis has been reported to be closely associated with systemic *Klebsiella pneumoniae* bacteremia and liver abscess. *C. albicans* is discovered as one of the most common pathogens in the endophthalmitis caused by fungal infection, whose treatment protocols include systemic and topical antifungal drugs, for instance, amphotericin B, voriconazole, and fluconazole.

On the other hand, studies about the treatment for bacterial inflammation are included in the red part, where glucocorticoids and a variety of antibiotics are mentioned, including vancomycin, gentamicin, ceftazidime, ciprofloxacin, levofloxacin, and moxifloxacin, which are frequently used alone or in combination depending on the clinical condition and the result of the gram stain of cultured bacteria. Moreover, drug susceptibility and antibiotic resistance of bacteria, and retinal toxicity of injections are also a concern (**Figure 4A**).

Besides the keyword co-occurrence network, CiteSpace’s keyword bursts analysis function is also a powerful tool for understanding the evolution and development trend. By presenting a concise linear relationship, we can quickly identify research hotspots during a specific period in the entire timespan. As displayed in **Figure 4B**, the top 25 keywords with the most robust citation bursts can be categorized as two phases according to their bursting year.

Before 2000, the related terms were mainly associated with cataract extraction. ECCE, which gradually replaced intracapsular cataract extraction (ICCE) with or without intraocular lens implantation, became the dominant procedure of that era (Verbraeken, 1993). Pseudophakic endophthalmitis was a common complication. Delayed-onset pseudophakic endophthalmitis is generally caused by less virulent *P. acnes* infection and is often tricky to treat (Al-Mezaine et al., 2009). Vitrectomy and intravitreal antimicrobials, including ciprofloxacin (antibacterial), amphotericin B, and fluconazole (antifungal), consistent with the co-occurrence trend above, have been proven to be effective measures (Durand, 2013).

However, after entering the 21st century, with the approval of intravitreal injection of anti-VEGF medications by the US Food and Drug Administration (FDA) for the treatment of neovascular age-related macular degeneration (AMD) in 2004 (Durand, 2017), the incidence of injection-related endophthalmitis, another mainstream manifestation, has gradually increased. Macular degeneration and anti-VEGF drugs (e.g., bevacizumab and ranibizumab) have received attention since 2010 (**Figure 4B**). After that, the controversy about causative organisms and the use of prophylactic antibiotics emerged and sparked heated debate. Intriguingly, although the term “endogenous endophthalmitis” appeared on the timeline from 1994, it has gained real prominence in recent years (2020-2023), probably due to its extremely low incidence.

### 3.7 Analysis of co-cited references

#### 3.7.1 Top co-cited references

References provide the knowledge basis and theoretical framework for subsequent research, and citation of references is crucial for scientific investigation. The fundamental theory of co-cited reference analysis lies that, supposing that two references are simultaneously cited by one literature, a “co-citation” behavior will be recognized, indicating the relevance between the references. In this case, the higher the co-citation frequency, the greater the reference value of the publication. Therefore, the highly co-cited articles are instructive for later studies in the field. **Table 5** counts the top 8 co-cited references, among which a review article that offered a comprehensive description of bacterial and fungal endophthalmitis in 2017 is far ahead in the citation count (Durand, 2017). Then, most of the remaining publications concern post-cataract endophthalmitis, led by a prospective, randomized, and multicenter clinical trial initiated by the ESCRS endophthalmitis study group (Barry and Grp, 2007). The designed trial compared intracameral cefuroxime at the end of surgery and perioperative levofloxacin drops for the prevention of postoperative endophthalmitis, and logistic regression analysis was used to identify several related risk factors, which deepened the understanding of endophthalmitis after cataract extraction (phacoemulsification with intraocular lens implantation).

**Table 5:** Top 8 co-cited references.

Rank	Citation counts	Author	Reference title	Journal	Year
1	90	Durand ML	Bacterial and fungal endophthalmitis	*Clin Microbiol Rev*	2017
2	57	Barry P	Prophylaxis of postoperative endophthalmitis following cataract surgery: Results of the ESCRS multicenter study and identification of risk factors	*J Cataract Refr Surg*	2007
3	56	Taban M	Acute endophthalmitis following cataract surgery - A systematic review of the literature	*Arch Ophthalmol-Chic*	2005
4	55	McCannel CA	Meta-analysis of endophthalmitis after intravitreal injection of anti-vascular endothelial growth factor agents - Causative organisms and possible prevention strategies	*Retina-J Ret Vit Dis*	2011
5	54	Friling E	Six-year incidence of endophthalmitis after cataract surgery: Swedish national study	*J Cataract Refr Surg*	2013
6	48	West ES	The incidence of endophthalmitis after cataract surgery among the US medicare population increased between 1994 and 2001	*Ophthalmology*	2005
7	46	Ciulla TA	Bacterial endophthalmitis prophylaxis for cataract surgery - An evidence-based update	*Ophthalmology*	2002
8	45	Jackson TL	Endogenous bacterial endophthalmitis: A 17-year prospective series and review of 267 reported cases	*Surv Ophthalmol*	2003

#### 3.7.2 Ten clusters of the co-citation network and cluster dependencies

**Figure 5:**
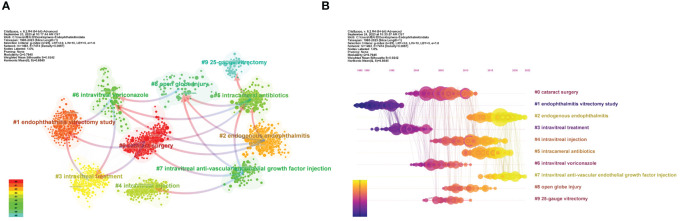
The main co-citation clusters. **(A)** CiteSpace visualization clusters of the co-cited references. Terms from the title field of the citing papers within each cluster are adopted as the definition of that cluster. **(B)** Timeline view of the listed clusters of the co-cited references. From: CiteSpace, v. 6.2.R4 (64-bit) Advance.

Based on the log-likelihood ratio (LLR) algorithm which can effectively process large-scale data and high-dimensional features and is generally applied to classification and model selection issues in various fields, CiteSpace can carve all references into several separate clusters, and papers of the same cluster represent a subtopic whose definition derives from the title terms of the citing papers in this cluster. **Figure 5A** retains the top 10 clusters, which are #0 cataract surgery, #1 endophthalmitis vitrectomy study, #2 endogenous endophthalmitis, #3 intravitreal treatment, #4 intravitreal injection, #5 intracameral antibiotics, #6 intravitreal voriconazole, #7 intravitreal anti-vascular endothelial growth factor injection, #8 open globe injury, and #9 25-gauge vitrectomy, respectively.

In the co-citation network, the arrows that connect clusters are called “cluster dependencies”. Like the dual-map overlay of journals, cluster dependencies represent the referential relationship between clusters, with arrows pointing to the cited cluster. Briefly, the left clusters have been tested over time and become a classic theoretical foreshadowing in the field, while the clusters on the right generally represent the latest advances and quote the published articles from the left clusters. From **Figure 5A**, not only can we discover close interaction among all the clusters, but the affiliation and knowledge extension between different clusters can also be intuitively visualized.

#### 3.7.3 Timeline map of clusters

The cluster map could be converted into a timeline view to record each cluster’s emergence, evolution, and development on the timeline (**Figure 5B**). Like cluster dependencies mentioned above, a timeline diagram can also vividly depict the links in the time dimension, reflected in the lines connecting the nodes. The EVS (cluster #1) is the first to emerge and afford support for subsequent intravitreal therapy (cluster #3). Also worth noting is that cataract surgery (cluster #0) became a hot academic topic in 2000-2010 and was linked to multiple clusters. It contains several papers with frequent co-citation (large nodes) and high betweenness centrality (marked purple ring). However, with advances in aseptic operational techniques and modified phacoemulsification, post-cataract endophthalmitis has declined dramatically. Currently, endogenous endophthalmitis (cluster #2), endophthalmitis after intravitreal anti-VEGF (clusters #4 and #7), and intracameral prophylactic antibiotics (cluster #5) are the subjects more discussed.

#### 3.7.4 Papers with high betweenness centrality

In the timeline view (**Figure 5B**), some nodes surrounded by purple rings can be observed, representing studies with a high betweenness centrality, which often act as a bridge between different sub-directions. Among these ten clusters, nine references with the highest betweenness centrality are included in **Table 6**. On the one hand, the incidence of postoperative endophthalmitis, especially following cataract surgery, was repeatedly introduced. Two studies were from the US (Aaberg et al., 1998; West et al., 2005), while the other presented data from the German population (Schmitz et al., 1999). On the other hand, the effectiveness of prophylactic antibiotic use after cataract surgery and intravitreal injection was evaluated (Ciulla et al., 2002; Garcia-Saenz et al., 2010; Bhatt et al., 2011). For example, a Spanish study compared the endophthalmitis rates in cataract surgery before and after using prophylactic intracameral cefuroxime. It concluded that cefuroxime effectively reduced the risk for acute-onset postoperative endophthalmitis (Garcia-Saenz et al., 2010).

**Table 6:** Cited references with the highest “betweenness centrality” among the top 10 clusters.

Rank	Centrality	References	Cluster #
1	0.32	West et al. (2005) The incidence of endophthalmitis after cataract surgery among the US medicare population increased between 1994 and 2001	0
2	0.28	Ciulla et al. (2002) Bacterial endophthalmitis prophylaxis for cataract surgery - An evidence-based update	0
3	0.16	Kresloff et al. (1998) Endophthalmitis	3
4	0.14	Aaberg et al. (1998) Nosocomial acute onset postoperative endophthalmitis survey - A 10-year review of incidence and outcomes	3
5	0.14	Garcia-Saenz et al. (2010) Effectiveness of intracameral cefuroxime in preventing endophthalmitis after cataract surgery ten-year comparative study	0
6	0.13	Major et al. (2010) Staphylococcus aureus endophthalmitis: antibiotic susceptibilities, methicillin resistance, and clinical outcomes	0
7	0.11	Bhatt et al. (2011) Prophylactic antibiotic use after intravitreal injection - Effect on endophthalmitis rate	4
8	0.11	Schmitz et al. (1999) Endophthalmitis in cataract surgery - Results of a German survey	3
9	0.11	Campochiaro (1994) Aminoglycoside toxicity in the treatment of endophthalmitis	1

#### 3.7.5 Details of cluster #1 (endophthalmitis vitrectomy study) and #9 (25-gauge vitrectomy)

Clusters #1 (endophthalmitis vitrectomy study) and #9 (25-gauge vitrectomy) are both related to vitrectomy (**Table 7**), with the difference that #1 explores the therapeutic effects of vitrectomy while #9 is concerned with endophthalmitis following PPV. In 1995, a randomized clinical trial named Endophthalmitis Vitrectomy Study (EVS) was conducted through 2×2 factorial analysis (immediate pars plana vitrectomy/vitreous tap or biopsy, with or without systemic antibiotics). This study found that vitrectomy benefited bacterial endophthalmitis patients with only light perception vision. On the contrary, routine immediate PPV may not be necessary when the vision is better than light perception (Vine et al., 1995). Although this trial has been carried out for a long time, its results are recognized as a guide for follow-up research (Han et al., 1996a, Han et al., 1996b; Johnson et al., 1997; Ng et al., 2005; Grzybowski et al., 2018). Regarding postoperative endophthalmitis caused by vitrectomy, which usually occurs within 15 days after the transconjunctival sutureless operation (Scott et al., 2008), the patient’s visual outcome is often poor, albeit with a low incidence (Eifrig et al., 2004; Kunimoto and Kaiser, 2007). On this basis, retrospective statistics indicated that 25-gauge vitrectomy was more likely to contribute to endophthalmitis than 20-gauge vitrectomy (Taban et al., 2006; Chen, 2007; Kunimoto and Kaiser, 2007; Scott et al., 2008).

**Table 7:** Cited references and citing articles of cluster #1 endophthalmitis vitrectomy study and #9 25-gauge vitrectomy.

Clusters	Cited references		Citing articles	
	Author (year) journal, volume	Citation counts	Author (year) title	Coverage counts
#1 Endophthalmitis vitrectomy study				
	Vine et al. (1995) *Arch Ophthalmol-Chic*, 113	44	Kresloff et al. (1998) Endophthalmitis	41
	Kattan et al. 1991 *Ophthalmology*, 98	35	Bron (1996) Endophthalmitis. 2: treatment	34
	Irvine et al. (1992) *Arch Ophthalmol-Chic*, 110	27	Meier and Wiedemann (1997) Endophthalmitis-clinical appearance, therapy and prevention	30
#9 25-gauge vitrectomy				
	Kunimoto and Kaiser (2007) *Ophthalmology*, 114	22	Bahrani et al. (2010) Endophthalmitis in the era of small gauge transconjunctival sutureless vitrectomy-meta analysis and review of literature	12
	Eifrig et al. (2004) *Am J Ophthalmol*, 138	20	Oshima et al. (2010) Multicenter survey with a systematic overview of acute-onset endophthalmitis after transconjunctival microincision vitrectomy surgery	12
	Scott et al. (2008) *Retina-J Ret Vit Dis*, 28	19	Chen (2007) 25-gauge transconjunctival sutureless vitrectomy	11

#### 3.7.6 Details of cluster #0 (cataract surgery), #4 (intravitreal injection), #7 (intravitreal anti-vascular endothelial growth factor injection), and #8 (open globe injury)

Providing that the outside microorganisms have access to entering the sterile intraocular tissue through openings on the ocular surface caused by various reasons, relatively common exogenous endophthalmitis may be triggered. According to the wound type, it can be subdivided into postoperative (cluster #0 cataract surgery), post-injection (cluster #4 intravitreal injection and #7 intravitreal anti-vascular endothelial growth factor injection), and post-traumatic (cluster #8 open globe injury) endophthalmitis.

Observably, most of the top co-cited papers (**Table 5**) are from cluster #0 (**Table 8**), showing that a lot of effort and attention has been spent on post-cataract endophthalmitis, including aspects of the incidence, risk factors, possible mechanisms, precautions, and dosing regimens (Ciulla et al., 2002; Taban et al., 2005; West et al., 2005; Barry and Grp, 2007). In addition to the previously mentioned ESCRS multicenter study, a systematic review also summarized the significant effect of incision type on the inflammatory risk, proposing that the incidence in the clear corneal incision group was 2.55 and 3.06 times (relative risk, RR) higher than the scleral incision and limbal incision group, respectively (Taban et al., 2005). The same concern was reviewed in a leading citing publication from this cluster (**Table 8**) (Lundstrom, 2006). Another study based on the US Medicare population concluded that individuals of older age and black race were at increased risk of post-cataract endophthalmitis between 1994 and 2001 (West et al., 2005). On the other hand, several citing articles evaluated the effectiveness of different antibiotic combinations for preventing and treating postoperative endophthalmitis (Busbee, 2006; O'brien et al., 2007; Fintelmann and Naseri, 2010).

**Table 8:** Cited references and citing articles of cluster #0 cataract surgery, #4 intravitreal injection, #7 intravitreal anti-vascular endothelial growth factor injection, and #8 open globe injury.

Clusters	Cited references		Citing articles	
	Author (year) journal, volume	Citation counts	Author (year) title	Coverage counts
#0 Cataract surgery				
	Barry and Grp (2007) *J Cataract Refr Surg*, 33	57	Fintelmann and Naseri (2010) Prophylaxis of postoperative endophthalmitis following cataract surgery: current status and future directions	20
	Taban et al. (2005) *Arch Ophthalmol-Chic*, 123	56	O'brien et al. (2007) Perspectives on antibiotics for postoperative endophthalmitis prophylaxis: potential role of moxifloxacin	20
	West et al. (2005) *Ophthalmology*, 112	48	Lundstrom (2006) Endophthalmitis and incision construction	17
	Ciulla et al. (2002) *Ophthalmology*, 109	46	Busbee (2006) Endophthalmitis: a reappraisal of incidence and treatment	17
#4 Intravitreal injection				
	Mccannel (2011) *Retina-J Ret Vit Dis*, 31	55	Moshfeghi (2011) Endophthalmitis following intravitreal anti-vascular endothelial growth factor injections for neovascular age-related macular degeneration	25
	Storey et al. (2014) *Ophthalmology*, 121	37	Schwartz et al. (2015) Controversies in topical antibiotics use with intravitreal injections	25
	Moshfeghi et al. (2011) *Retina-J Ret Vit Dis*, 31	37	Brynskov et al. (2014) No cases of endophthalmitis after 20,293 intravitreal injections in an operating room setting	24
#7 Intravitreal anti-vascular endothelial growth factor injection				
	Pershing et al. (2020) *Ophthalmology*, 127	33	Singh et al. (2022) Preventive factors, diagnosis, and management of injection-related endophthalmitis: a literature review	19
	Xu et al. (2018) *Ophthalmology*, 125	30	Patel et al. (2022) Complications of intravitreal injections: 2022	10
	Soliman et al., (2019) *Ophthalmol Retina*, 3	16	Patel et al. (2021a) The impact of physician face mask use on endophthalmitis after intravitreal anti-vascular endothelial growth factor injections	8
#8 Open globe injury				
	Chiquet et al. (2008) *Invest Ophth Vis Sci*, 49	25	Chhablani (2011) Fungal endophthalmitis	8
	Bhagat et al. (2011) *Surv Ophthalmol*, 56	22	Yang et al. (2011) Intravitreally implantable voriconazole delivery system for experimental fungal endophthalmitis	7
	Chakrabarti et al. (2008) *Retina-J Ret Vit Dis*, 28	17	Joseph et al. (2012) Real-time polymerase chain reaction in the diagnosis of acute postoperative endophthalmitis	7

Clusters #4 and #7 are the same in terms of content, and they are both about possible intraocular inflammation after intravitreal anti-VEGF (**Table 8**). With the gradual widespread use of anti-VEGF, post-injection endophthalmitis has begun to attract attention. Contributions in these two clusters concentrated on incidence, visual outcomes, and pathogenic microbial profiles. Although the incidence was low (varying from 0.015% to 0.05% reported in different studies) (Mccannel, 2011; Moshfeghi et al., 2011; Storey et al., 2014), visual acuity outcome was generally unsatisfactory and associated with more common and severe *streptococcal* infections (Mccannel, 2011; Moshfeghi, 2011; Moshfeghi et al., 2011; Xu et al., 2018). Therefore, avoiding treatment on eyes with active ocular surface or eyelid diseases and reducing droplet transmission during the injection, such as avoiding talking, coughing, sneezing, and wearing a medical mask, may be practical measures (Mccannel, 2011; Patel et al., 2021a; Singh et al., 2022).

Open globe injury (cluster #8) is a broad type of ocular trauma characterized by full-thickness lacerations of the cornea and sclera, either penetrating or perforating (Kuhn et al., 2004). According to the US Eye Injury Registry report, 3.4% of open globe injuries had endophthalmitis (Danis, 2002). Moreover, because the pathogens causing post-traumatic endophthalmitis are distinct from those in other types (Bhagat et al., 2011), this topic forms a separate cluster (**Table 8**). Regular pathogens are coagulase-negative *Staphylococci*, *B. cereus* (Hong et al., 2016), and sometimes fungi (most commonly *C. albicans* and *Aspergillus*) (Chakrabarti et al., 2008; Chhablani, 2011). Microorganisms with different virulence could directly influence visual prognosis. Intravenous and intravitreal antibiotic treatment should be started urgently (Bhagat et al., 2011).

#### 3.7.7 Details of cluster #2 (endogenous endophthalmitis) and #6 (intravitreal voriconazole)

Accounting for only 5% to 15% of all endophthalmitis cases, endogenous endophthalmitis is blood-borne and in most cases associated with severe systemic infection. Approximately 0.05% to 0.4% fungemia and 0.04% bacteremia may be complicated with endophthalmitis (Spelta et al., 2021). Of note, in cluster #2 (endogenous endophthalmitis), two reviews written by Durand ML are recognized as the popular co-cited papers (**Table 9**), which both detailed a variety of endogenous and exogenous endophthalmitis, either bacterial or fungal infections, and updated the research progress on this rare but severe disease (Durand, 2013, Durand, 2017). In particular, the review article published in 2017 is the most cited reference in all clusters (**Table 5**) (Durand, 2017). Additionally, in the list of the cited articles with the most coverage, Danielescu C et al. (Danielescu et al., 2020, Danielescu et al., 2022). recently reviewed an endogenous endophthalmitis case series and the diagnosis and treatment of fungal endophthalmitis. Blood cultures revealed that hematogenous disseminated infection with fungi such as *Candida* was a major cause of endogenous endophthalmitis. Thus, as one of the significant quotes of cluster #2 (**Figure 5A**), cluster #6 (intravitreal voriconazole) involves the connections among the epidemiology, cultured microorganisms and visual acuity outcomes of endogenous endophthalmitis (Jackson et al., 2003; Schiedler et al., 2004; Ness et al., 2007), along with the role of intravitreal anti-fungal voriconazole in the management of culture-proven endophthalmitis (**Table 9**) (Breit et al., 2005; Yang et al., 2011).

**Table 9:** Cited references and citing articles of cluster #2 endogenous endophthalmitis and #6 intravitreal voriconazole.

Clusters	Cited references		Citing articles	
	Author (year) journal, volume	Citation counts	Author (year) title	Coverage counts
#2 Endogenous endophthalmitis				
	Durand (2017) *Clin Microbiol Rev*, 30	90	Tranos et al. (2016) Current perspectives of prophylaxis and management of acute infective endophthalmitis	32
	Relhan et al. (2018) *Am J Ophthalmol*, 187	42	Danielescu et al. (2022) The diagnosis and treatment of fungal endophthalmitis: an update	27
	Durand (2013) *Clin Microbiol Infec*, 19	36	Danielescu et al. (2020) Endogenous endophthalmitis: a review of case series published between 2011 and 2020	26
#6 Intravitreal voriconazole				
	Jackson et al. (2003) *Surv Ophthalmol*, 48	45	Yang et al. (2011) Intravitreally implantable voriconazole delivery system for experimental fungal endophthalmitis	12
	Breit et al. (2005) *Am J Ophthalmol*, 139	23	Chiquet et al. (2007) Acute endophthalmitis: from bacteria to clinical settings	9
	Schiedler et al. (2004) *Am J Ophthalmol*, 137	22	Ness et al. (2007) Endogenous endophthalmitis: microorganisms, disposition and prognosis	8

#### 3.7.8 Details of cluster #3 (intravitreal treatment) and #5 (intracameral antibiotics)

Finally, clusters #3 (intravitreal treatment) and #5 (intracameral antibiotics) refer to the application of antibiotics to treat or prevent acute-onset postoperative bacterial endophthalmitis (**Table 10**). Administration of intravitreal antibiotics is the mainstay of treatment for acute-onset endophthalmitis and achieves higher intraocular antibiotic concentrations than any other modality of administration (Kresloff et al., 1998). The EVS found that all gram-positive isolates were susceptible to vancomycin, while most isolated gram-negative organisms were equally sensitive to amikacin and ceftazidime (Han et al., 1996b). On the side, following the clear benefits of intracameral cefuroxime reported by prospective ESCRS study (Barry and Grp, 2007), multiple data highlighted the inhibitory effects of intracameral antibiotic prophylaxis on postoperative endophthalmitis, where cefuroxime, vancomycin, and moxifloxacin were preferred (Barry, 2014; Chang et al., 2015; Haripriya, 2017; Haripriya et al., 2017). In a Swedish national study, the non-use of intracameral cefuroxime was even identified by logistic regression as a significant risk factor of endophthalmitis (Friling et al., 2013).

**Table 10:** Cited references and citing articles of cluster #3 intravitreal treatment and #5 intracameral antibiotics.

Clusters	Cited references		Citing articles	
	Author (year) journal, volume	Citation counts	Author (year) title	Coverage counts
#3 Intravitreal treatment				
	Han et al. (1996b) *Am J Ophthalmol*, 122	44	Montan (2001) Endophthalmitis	25
	Aaberg et al. (1998) *Ophthalmology*, 105	31	Callegan et al. (2002) Bacterial endophthalmitis: epidemiology, therapeutics, and bacterium-host interactions	23
	Callegan et al. (2002) *Clin Microbiol Rev*, 15	31	Jackson et al. (2003) Endogenous bacterial endophthalmitis: a 17-year prospective series and review of 267 reported cases	13
#5 Intracameral antibiotics				
	Friling et al. (2013) *J Cataract Refr Surg*, 39	54	Haripriya (2017) Antibiotic prophylaxis in cataract surgery - an evidence-based approach	34
	Chang et al. (2015) *J Cataract Refr Surg*, 41	38	Kuklo et al. (2017) Hot topics in perioperative antibiotics for cataract surgery	31
	Haripriya et al. (2017) *Ophthalmology*, 124	34	Garg et al. (2017) Endophthalmitis after cataract surgery: epidemiology, risk factors, and evidence on protection	24

## 4 Discussion

As a dangerous eye disease, endophthalmitis has been universally concerned—an increasing number of papers have been published, and a relatively mature system has formed. Nevertheless, despite the advent of a brief bibliographic review in 2021 (Wade et al., 2021), there has been a lack of systematic historical combing and bibliometric analyses in the field. This study is the first bibliometric study and visualization analysis that adopts CiteSpace and VOSviewer to construe all endophthalmitis-related documents from the WOS over the last three decades.

### 4.1 Brief historical retrospection of research on endophthalmitis

The understanding of endophthalmitis has undergone a long period of transition. It can be roughly divided into three phases: the pre-antimicrobial era (before the 1940s), the predominantly systemic antimicrobial era (mid-1940s to early 1970s), and the current intravitreal antimicrobial era (early 1970s to now) that studies included in our analysis belong to (Relhan et al., 2018). During the pre-antimicrobial era, therapies were reported, including antiserum administration, aqueous mercurochrome drops, topical heating, or intramuscular injection of boiled milk (Haden, 1918). Subsequently, the frequency of systemic and adjunctive topical antimicrobials increased, and better therapeutic outcomes were acquired, yet this was accompanied by more extended hospitalization, usually five days or more (Allen and Mangiaracine, 1964). With the deepening of experiments and improved medicines, intravitreal antimicrobial injection was perceived as the standard therapeutics for clinically suspected endophthalmitis in the late 1970s and has become a vital component of the treatment of endophthalmitis today (Flynn and Scott, 2008). It is confirmed by clusters #3 (intravitreal treatment) and #6 (intravitreal voriconazole) discussed in our article (**Figures 5A, B**). As **Figure 2A** shows, there has been no shortage of endophthalmitis-related publications since 1993, with an apparent upward trend during recent years. Also, the gradually elevated citation counts indicate that ophthalmology practitioners increasingly value this field. Among those records, the landmark studies are the EVS, the ESCRS multicenter study, and the American Society of Cataract and Refractive Surgery (ASCRS) member survey.

#### 4.1.1 Endophthalmitis vitrectomy study

To alleviate the social burden of the alarming postoperative complication, the EVS group was established in the United States in the late 1980s to conduct the Endophthalmitis Vitrectomy Study (EVS). The group recruited 420 patients between February 1990 and January 1994, and aimed to evaluate the role of PPV and systemic antibiotics in acute-onset endophthalmitis following cataract surgery or secondary intraocular lens implantation (Vine et al., 1995). As the only multicenter, prospective, randomized clinical trial of vitrectomy to date, the EVS compared the efficacy of PPV and tap/biopsy in the management of post-cataract endophthalmitis and was dedicated to reasonable treatment guidelines (Vine et al., 1995; Grzybowski et al., 2018), thus laying the groundwork for the leading position of the United States in this field (**Table 1**; **Figure 2C**). It is worth noting that the two core co-cited authors, Han DP and Vine AK (**Table 2**; **Figure 2D**), are both from the EVS group. Because the primary observed outcome was the recoverable visual acuity after 9-12 months, the vital conclusion was that the benefit from vitrectomy over tap/biopsy was admitted in patients with only light perception vision, while no advantages were presented in patients with better visual acuity than delicate perception (Vine et al., 1995). Meanwhile, the utilization of systemic ceftazidime and amikacin exerted no direct alterations on final visual acuity or media clarity, so omitting intravenous antibiotics could reduce side effects and help control the cost and duration of hospital stay (Vine et al., 1995).

Additionally, during the following years (notably 1996-2001), the study team handled this prospective data to supplement a series of additional analyses (Han et al., 1996a, Han et al., 1996b; Bannerman et al., 1997; Barza et al., 1997; Johnson et al., 1997; Wisniewski et al., 1997; Doft et al., 1998; Han et al., 1999; Doft et al., 2000; Wisniewski et al., 2000; Doft et al., 2001), which were mainly published in several authoritative ophthalmology journals, including *Archives of Ophthalmology* (the predecessor of *JAMA Ophthalmology*), *Ophthalmology*, and *AJO* (**Table 3**; **Figure 3B**). Some recommendations or perspectives were given.

First, reduced visual acuity, conjunctival hyperemia, pain, hypopyon, and eyelid swelling were found to be typical clinical presentations of post-cataract endophthalmitis, and these features helped predict a possible cultured microbiologic spectrum (Johnson et al., 1997; Wisniewski et al., 2000). Of all intraocular sample isolates obtained, 70% were gram-positive coagulase-negative *Staphylococci* with *S. epidermidis* predominantly, which were identified to potentially originate from periocular skin flora by pulsed-field gel electrophoresis, reinforcing the essentiality for rigorous preoperative disinfection of the surgical sites (Han et al., 1996b; Bannerman et al., 1997).

Second, concerning the diagnostic techniques, vitrectomy cassette fluid did not have a higher culture-positive rate than undiluted vitreous obtained by tap/biopsy (Barza et al., 1997). Similarly, there was no significant difference between mechanized vitreous biopsy and needle aspiration regarding microbiologic yields and operative complication rates in EVS (Han et al., 1999).

Third, although visual prognosis is closely related to the type of organisms and their gram stain results, baseline visual acuity at initial diagnosis was put in a better position than microbiological factors in predicting visual outcome and determining immediate vitrectomy value (Han et al., 1996a). Moreover, patients who required additional procedures due to surgical complications and worsening intraocular infections or who developed retinal detachment after the initial treatment generally owned much worse follow-up visual acuity, and these factors were seen as a sign of more severe disease (Doft et al., 1998, Doft et al., 2000).

Fourth, diabetes mellitus, a common systemic disease, was studied separately. It was reported that diabetes seemed to be relevant to coagulase-negative *micrococcal* infection (Johnson et al., 1997). For diabetic patients with initial visual acuity better than light perception, the proportion achieving visual acuity of 20/40 after vitrectomy (57%) was slightly higher than that after tap/biopsy (40%). However, no statistical difference was figured out, which means the optimal treatment regimen for diabetic patients needs to be further clarified (Doft et al., 2001).

Finally, the economic implications of EVS were also taken into account, which was one of the original intentions of the study (Flynn and Scott, 2008). The charge-effectiveness analysis in 1996 estimated that assuming the EVS recommendations were followed for endophthalmitis after cataract surgery, the annual cost of hospitalization in the United States would fall by between $7.6 million and $40.0 million (Wisniewski et al., 1997).

Voices of skepticism remained. The most prominent criticism was the choice of ceftazidime and amikacin as intravenous antibiotics for post-cataract endophthalmitis, as they have poor activity against the most commonly isolated *staphylococci* in EVS and the inability of amikacin to cross the blood-eye barrier results in minimal intraocular concentrations (Durand, 2013). A western Australian report suggested that despite significant changes in managing postoperative endophthalmitis since the EVS, patients’ visual outcomes did not improve, which was connected to a lack of oral antibiotic therapy (Ng et al., 2005).

On the other hand, the indications for implementing PPV were also controversial. The 2002 Canadian survey showed that most Canadian vitreoretinal surgeons did not strictly follow the recommendations of EVS (Siddiqui et al., 2002). The benefits of PPV may be underestimated in the EVS due to the exclusion of severe cases with anterior chamber opacification or without light perception (Flynn and Scott, 2008), along with the absence of subjects with other types of endophthalmitis caused mainly by more virulent organisms (e.g., *streptococcus*). What’s more, considering that nearly 30 years have passed since the release of the EVS, during which significant progress has been made on vitrectomy, and the minimally invasive surgery allows doctors to minimize operative risks and achieve better outcomes, some researchers insist on complete and early vitrectomy for endophthalmitis (Grzybowski et al., 2018). In short, treating acute postoperative endophthalmitis should not solely focus on initial visual acuity but also consider individualized clinical manifestations and disease duration.

#### 4.1.2 ESCRS multicenter study and ASCRS member survey

Unlike EVS, the ESCRS study and ASCRS survey centered on acute post-cataract endophthalmitis prophylactic approaches. All their results were published in *JCRS*, an influential journal of cataract and refractive surgery (**Table 3**). Barry P (the ranking co-cited author in **Table 2**) led the implementation of the ESCRS multicenter study, which was finished together by 24 ophthalmology units in Austria, Belgium, Germany, Italy, Poland, Portugal, Spain, Turkey, and the United Kingdom, so it is not surprising that the United Kingdom becomes the other country with a high centrality outside of the United States (**Table 1**; **Figure 2C**). This European study fully affirmed the significant preventive effect of intracameral cefuroxime on post-cataract endophthalmitis at the conclusion of surgery. A lack of intracameral cefuroxime prophylaxis would result in a near five-fold risk of endophthalmitis (Barry et al., 2006; Seal et al., 2006; Barry and Grp, 2007). Concerning laboratory diagnostics, the accessional studies noted that the introduction of molecular biology techniques like polymerase chain reaction (PCR) improved the identification rate of pathogens, with a statistical association between the laboratory-proven endophthalmitis cases and clinical symptoms and signs, including eyelid swelling, pain, and vitreous opacity (Seal et al., 2008; Barry et al., 2009). Subsequent surveys towards European ophthalmic surgeons demonstrated that adopting intracameral prophylactic antibiotics became mainstream (Gore et al., 2009; Barry, 2014). Likewise, retrospective evidence from Portugal and Italy supported the protective effect of cefuroxime against endophthalmitis (Beselga et al., 2014; Grosso et al., 2016).

The prevalence of intracameral antibiotics is also reflected in the changeover in the results of the ASCRS member surveys. ASCRS launched three online surveys to its members in 2007 (Chang et al., 2007), 2014 (Chang et al., 2015), and 2021 (Chang and Rhee, 2022), aiming to understand the intentions of refractive surgeons regarding prophylactic antibiotic regimens. Right after the publication of the ESCRS results, the 2007 ASCRS questionnaire indicated a solid tendency to topically use the latest generation of fluoroquinolones, with no use of the intracameral form in a whopping 77% of the respondents (Chang et al., 2007). From 50% in 2014 to 66% in 2021, the anterior chamber injection of prophylactic antibiotics gradually replaced topical agents and was transformed into a consensus under the influence of the ESCRS research series, in which vancomycin utilization gradually declined in the United States, followed by more frequent injections of moxifloxacin (Chang et al., 2015; Chang and Rhee, 2022). Besides, commercially approved antibiotic formulations for intracameral prophylaxis are highly anticipated to avoid potential dilution and contamination risks.

### 4.2 Diverse pathogenic microorganisms contribute to infectious endophthalmitis

In the analyses of keywords (**Figures 4A, B**) and references (**Figures 5A, B**; **Tables 8**, **9**), the classification of infectious endophthalmitis, both exogenous and endogenous endophthalmitis, has been detailed elucidated, with their featured pathogenic microorganisms and lesion characteristics. Another broadly adopted classification principle is in light of the species of causative organisms.

#### 4.2.1 Bacterial endophthalmitis

##### 4.2.1.1 Coagulase-negative *Staphylococci*

Gram-positive coagulase-negative *Staphylococci*, particularly *S. epidermidis*, are the most common isolates of exogenous endophthalmitis. As the prominent component of the periocular skin flora, coagulase-negative *Staphylococci* play a significant role in intraocular infections. Contaminated instruments, irrigating fluids, implants, and IOFB make it easy for *Staphylococci* to gain access readily to the intraocular compartments at the time of open eye surgery, intravitreal injection, and open globe injuries, causing infection and inflammation (Bannerman et al., 1997; Bhagat et al., 2011; Xu et al., 2018). However, less virulent *Staphylococci*-related endophthalmitis is associated with milder manifestations and better visual prognosis than those of other infections (Xu et al., 2018). Apart from conventional intravitreal vancomycin as the initial treatment, preoperative disinfection of the ocular surface with a concentration of 5-10% povidone-iodine is also emphasized. It has been reported to effectively cut down bacteria on the conjunctiva and eyelid, and reduce the risk of introducing infectious organisms (Fintelmann and Naseri, 2010).

##### 4.2.1.2 Propionibacterium acnes

Delayed-onset or chronic postoperative endophthalmitis, defined as occurring six weeks or longer after surgery, is easy to overlook. *P. acnes* is the major pathogen that induces late but persistent intraocular infections, resulting in a frustrating visual prognosis and difficulty curing. If low-grade inflammation in the anterior chamber persistently exist, treatments with a combination of removal or exchange of the intraocular lens, intravitreal antibiotics, and vitrectomy will be required (Durand, 2013).

##### 4.2.1.3 Viridians Streptococci

*Viridians Streptococci*, also called as alpha hemolytic *Streptococci*, are a kind of important normal commensals, with the largest distribution in the oral cavity, such as *S. mitis* and *S. oralis*. Compared with postoperative condition, a much higher rate of *Streptococcal* endophthalmitis is presented after intravitreal injection and often associated with worse visual outcomes (Moshfeghi et al., 2011). Vancomycin plus ceftazidime can be used as the first-line agent. PPV is considered as an additional therapy when severe infection occurs (Xu et al., 2018).

##### 4.2.1.4 Bacillus cereus

For the more complex situation of post-traumatic endophthalmitis, *B. cereus*, coagulase-negative *Staphylococci*, *Streptococci*, and gram-negative species such as *Klebsiella* are predominant culture-positive bacteria (Long et al., 2014). Among them, the virulent *B. cereus* can lead to fulminant infections involving the eyeball and orbit, in which the infected patients are mostly companied with rapid disease progression. Symptoms like eye pain, redness, swelling, and vision loss often appear within 12 to 24 hours after trauma, giving rise to very poor vision acuity (Mei et al., 2021). Thus, treatment is supposed to be aggressive and initiated urgently with systemic and intravitreal antibiotics. In most cases, vitrectomy is necessary to clear the foci directly to control the refractory inflammation (Zheng et al., 2019).

#### 4.2.2 Fungal endophthalmitis

Fungal infections are more common in endogenous endophthalmitis, which originates from infections of other sites (e.g., liver abscess, endocarditis, and urinary tract infections) through blood spread, because of systemic immunodeficiency (Durand, 2017). The visual outcomes are incredibly pessimistic and unsatisfactory, with blindness in most cases, and extraocular foci are often associated with high mortality (Jackson et al., 2003; Schiedler et al., 2004). As a severe complication of systemic candidemia, *Candida* endophthalmitis has been identified as the most frequent form of endogenous fungal endophthalmitis, followed by mold endophthalmitis, including *Aspergillus* and *Fusarium* infections (Kramer et al., 2006; Yoshida et al., 2018; Zhuang et al., 2020). For therapeutic strategies, immediate intravitreal administration combined with systemic antifungal medications and subsequent vitrectomy is beneficial. Amphotericin B and voriconazole are commonly used antifungal agents and have exhibited favorable effects in controlling inflammation (Yang et al., 2011; Bae and Lee, 2015; Zhao et al., 2015; Bienvenu et al., 2020). What’s more, several case reports published in recent years have shown that in the cases of resistant fungal endophthalmitis, intravitreal caspofungin, the first approved antifungal echinocandin, can be an ideal and safe alternative to the former two (Danielescu et al., 2017; Yadav et al., 2017; Von Jagow et al., 2020; Nakhwa, 2021).

### 4.3 Emerging problems in the context of COVID-19

With the outbreak of COVID-19, endophthalmitis-related research has experienced a sharp increase since 2020 (**Figure 2A**). Some possible correlations between endophthalmitis and the COVID-19 pandemic were revealed.

For one thing, due to the weakened resistance to infection caused by COVID-19 and the widespread use of high-dose systemic corticosteroids to critically ill patients during treatment, the immune response has been greatly suppressed, which exacerbates fungal invasion and opportunistic infections in the absence of effective antifungal drugs (Bayram et al., 2021; Fayed et al., 2022). Several cases of endogenous fungal endophthalmitis recovered from or hospitalized for COVID-19 were reported, mainly about *Candida* and *Aspergillus* endophthalmitis (Shroff et al., 2021; Kaluarachchi and Abeykoon, 2022; Mehta et al., 2022; Fekri et al., 2023; Fossataro et al., 2023; Mohan et al., 2023). With no exception, these patients had been treated with prolonged systemic steroids, and were accompanied with certain systemic risk factors, such as type 2 diabetes.

For another, the effect of universal mask-wearing on the incidence of endophthalmitis is controversial. Oral commensal organisms, such as *Streptococcus*, can trigger intraocular infections, particularly endophthalmitis after intravitreal anti-VEGF injections in a germy environment. Blocking droplet transmission may help control infections with these bacteria. Thus, the implementation of universal masking under the epidemic can theoretically conduce to reduce the incidence of post-injection endophthalmitis. However, retrospective data did not show this trend, although a reduced culture-positive rate was figured out (Naguib et al., 2021; Patel et al., 2021b). Interestingly, a Japanese study published in the *British Journal of Ophthalmology* (*BJO*) reported an increased incidence of post-vitrectomy endophthalmitis with oral commensals, which are reportedly rare in endophthalmitis following vitrectomy, in the COVID-mask period (Sakamoto et al., 2022). Reasonable assumptions were made. For example, inappropriate face mask wearing allows exhaled air containing oral bacteria to flow from the upper part of the mask into the ocular surface, thereby increasing the risk of intraocular infection (Tanaka et al., 2022). Moreover, the study found a unique infection of *Staphylococcus lugdunensis* in the COVID-mask period. Because *S. lugdunensis* is resident on the back of the auricle, the possibility that hands might touch the auricle and spread the pathogen to periocular areas during mask-wearing was proposed (Sakamoto et al., 2022).

### 4.4 The limitations of the study

Nevertheless, there are some limitations to our study. First, since bibliometric analysis can solely focus on a single database, we have to select the authoritative SCI-Expanded database under the WOSCC, ignoring several useful medical databases, such as PubMed, whose additional documents may shed new light on our topic. Second, our research relies heavily on two computer software, Citespace and VOSviewer. Yet, there is always a slight deviation in the machine algorithm. For instance, clusters #4 (intravitreal injection) and #7 (intravitreal anti-vascular endothelial growth factor injection) in the co-cited cluster analysis (**Figure 5A**) are actually both about endophthalmitis caused by intravitreal injection of anti-VEGF, which are contiguous on the timeline (**Figure 5B**) but are grouped into two separate clusters. Last, endophthalmitis is indeed a large and intricate topic. As a result, it isn’t easy to consider all aspects in depth. Still, we are trying to elaborate as much as possible on the trends and priorities of research under this subject and systematically comb a series of landmark studies.

## 5 Conclusion

Based on previous studies, we are amid the intravitreal antimicrobial era with an explosion of research. The United States currently leads the way in this field, and European countries, including the United Kingdom, have also made significant contributions. Articles published in specialized journals such as *Ophthalmology* receive the most attention. For all categories of endophthalmitis, whether bacterial or fungal, endogenous or exogenous, the importance of timely and effective initial empiric treatment and subsequent individualized regimens based on cultured pathogens can never be ignored. With the progress of time, the prevention of endophthalmitis, such as intracameral cefuroxime and preoperative povidone-iodine, has gradually attracted the attention of ophthalmology researchers and physicians. Nonetheless, given the variability of pathogenic microorganisms, there is still a long way to go to overcome the drug resistance of bacteria and fungi and seek safe and effective alternatives.

## Data availability statement

The original contributions presented in the study are included in the article/supplementary material. Further inquiries can be directed to the corresponding author.

## Author contributions

XF: Conceptualization, Data curation, Formal analysis, Investigation, Methodology, Resources, Software, Validation, Visualization, Writing – original draft, Writing – review & editing. WD: Data curation, Investigation, Methodology, Software, Validation, Visualization, Writing – review & editing. LH: Data curation, Formal analysis, Investigation, Methodology, Software, Validation, Visualization, Writing – review & editing. XR: Data curation, Funding acquisition, Investigation, Validation, Writing – review & editing. DC: Conceptualization, Funding acquisition, Supervision, Writing – review & editing.
